# Autoimmune pancreatitis: A bibliometric analysis from 2002 to 2022

**DOI:** 10.3389/fimmu.2023.1135096

**Published:** 2023-02-22

**Authors:** Xian-Da Zhang, Yao Zhang, Yi-Zhou Zhao, Chun-Hua Zhou, Duo-Wu Zou

**Affiliations:** Department of Gastroenterology, Ruijin Hospital, Shanghai Jiao Tong University School of Medicine, Shanghai, China

**Keywords:** autoimmune pancreatitis, bibliometrics, CiteSpace, emerging topics, research focus, VOSviewer

## Abstract

**Background/Objectives:**

Autoimmune pancreatitis (AIP) is a distinct form of pancreatic inflammatory disease that responds well to glucocorticoid therapy. Knowledge on AIP has rapidly evolved over the past two decades. Based on bibliometric analysis, this study aimed to assess the research status of AIP over the past two decades and determine the research focus and emerging topics.

**Methods:**

AIP-related publications published between January 1, 2002, and June 6, 2022, were retrieved from the Web of Science Core Collection. Bibliometric data were analyzed using HisCite, VOSviewer, CiteSpace, and *bibliometrix* package. Annual output, leading countries/regions, active institutions and authors, core journals and references, and keywords of AIP were evaluated.

**Results:**

Overall, 1,772 publications were retrieved from 501 journals by 6,767 authors from 63 countries/regions. Japan published articles on AIP the most (n=728, 41.1%), followed by the United States (n=336, 19%), Germany (n=147, 8.3%), China (n=127, 7%), and Italy (n=107, 6%). The top three most prolific authors were Terumi Kamisawa from Tokyo Metropolitan Komagome Hospital (n=117), Kazuichi Okazaki from Kansai Medical University (n=103), and Shigeyuki Kawa from Matsumoto Dental University (n=94). *Pancreas* was the most productive journal regarding AIP research (n=95), followed by the *Journal of Gastroenterology* (n=67), *Internal Medicine* (n=66), *Pancreatology* (n=63), and *World Journal of Gastroenterology* (n=62). “Diagnosis” was the most mentioned keyword. “Risk,” “malignancy,” “outcome,” “22-gauge needle,” and “fine-needle aspiration” were recognized as emerging topics.

**Conclusion:**

Japan was the leading country in AIP research. Research papers were mainly published in specialized journals. Diagnosis was the research focus. Long-term outcomes and pancreatic tissue acquisition were recognized as research frontiers for AIP.

## 1 Introduction

Autoimmune pancreatitis (AIP) is a distinct form of pancreatic inflammatory disease that responds well to glucocorticoid therapy (1). AIP can be classified into types 1 and 2 based on clinical and pathological findings. Type 1 AIP is the pancreatic manifestation of IgG4-related disease (IgG4-RD), which is characterized by an elevation of serum IgG4 levels and infiltration of IgG4-positive plasmacytes (2, 3), whereas type 2 is more localized in the pancreas, with normal serum IgG4 levels and the presence of neutrophil infiltration (2, 3). As a relatively newly identified disease, the knowledge on the diagnosis, treatment, and clinical outcomes of AIP has rapidly evolved over the past two decades.

Bibliometric analysis enables the qualitative and quantitative profiling of publications (4) and allows researchers to identify not only the productive countries/regions, institutions, and authors but also the research focus and emerging topics within a specific field (5, 6). Additionally, bibliometric analysis has been applied in research on autoimmune digestive diseases, including inflammatory bowel disease and primary biliary cholangitis (7, 8). However, a bibliometric analysis of AIP has not been reported in the literature thus far.

In the current study, bibliometric analysis was utilized to assess the research status of AIP over the past two decades, as well as identify the research focus and emerging topics.

## 2 Materials and methods

### 2.1 Search strategy

Literature search was performed in the Web of Science Core Collection (WoSCC) on June 6, 2022, at the Ruijin Hospital affiliated to the Shanghai Jiao Tong University School of Medicine. Thesauruses of AIP were identified in the Medical Subject Headings (MeSH) database (https://www.ncbi.nlm.nih.gov/mesh) and added to the search query, as follows: TI = (“autoimmune pancreatitis” OR “IgG4-related pancreatitis” OR “lymphoplasmacytic sclerosing pancreatitis” OR “idiopathic duct centric pancreatitis”) OR AB = (“autoimmune pancreatitis” OR “IgG4-related pancreatitis” OR “lymphoplasmacytic sclerosing pancreatitis” OR “idiopathic duct centric pancreatitis”) OR AK = (“autoimmune pancreatitis” OR “IgG4-related pancreatitis” OR “lymphoplasmacytic sclerosing pancreatitis” OR “idiopathic duct centric pancreatitis”). According to our search query, articles that mentioned AIP or its synonyms in the title, abstract, or keywords were identified. The date of publications was set between January 1, 2002, and June 6, 2022, and the type of publications was restricted to articles and review articles. Documents published earlier than January 1, 2002, were excluded. Moreover, case reports, meeting abstracts, editorial materials, and other documents types were excluded. No restriction on languages was applied.

### 2.2 Data collection

Information on the literature identified by search query was downloaded from the WoSCC on June 6, 2022. Details of the literature, including author, title, source, sponsors, times cited count, accession number, abstract, address, document type, and cited references, were downloaded in txt and BibTex formats for further analysis. The H-index of the top 10 most productive authors were collected from Web of Science on June 6, 2022. The 2021 impact factor and 2021 Journal Citation Report category quartile of the top 10 core journals in AIP were collected from Web of Science.

### 2.3 Statistical analysis

Bibliometric data were analyzed using HisCite (version 12.03.17), VOSviewer (version 1.6.18), CiteSpace (version 6.1.R3), and *bibliometrix* package (version 3.2.1; https://cran.r-project.org/web/packages/bibliometrix/) based on R language (version 4.1.2). HisCite was used to identify the number of publications and the number of citations for productive countries, institutions, and authors. The top 10 publications with the highest number of citations in AIP research were recognized by HisCite. The annual number of publications was also identified by HisCite and visualized by *ggplot2* package (version 3.3.6; https://github.com/tidyverse/ggplot2) based on R language. VOSviewer was used to recognize the top 10 keywords with the highest number of occurrences, as well as the clustering of the top 50 keywords. A list of thesauri was employed for better understanding, which included “serum IgG4 concentrations,” represented by “serum IgG4”; “diagnostic criteria,” represented by “diagnosis”; “carcinoma,” represented by “cancer”; “disease,” represented by “IgG4-related disease”; “clinical feature”; and “characteristics,” represented by “features.” CiteSpace was used to construct a dual-map overlay of the journals related to AIP and to perform a keyword burst detection of the top 25 keywords with the strongest emergent strength. CiteSpace was used to measure the collaborative centrality of countries/regions, institutions, and authors. The setting of CiteSpace was as follows: scale factor k=25, the strength of links measured by cosine, the scope of links measured within slices, and pruning with pathfinder and sliced network. The distribution of publications and collaborations between countries/regions and the annual output of the top 10 most productive authors were visualized using *bibliometrix* package. Clustering of collaboration among countries/regions, institutions, and authors was also visualized by *bibliometrix* package. The ratios of original and review articles for each year were measured using *bibliometrix* package.

## 3 Results

### 3.1 Overview

**Figure 1:**
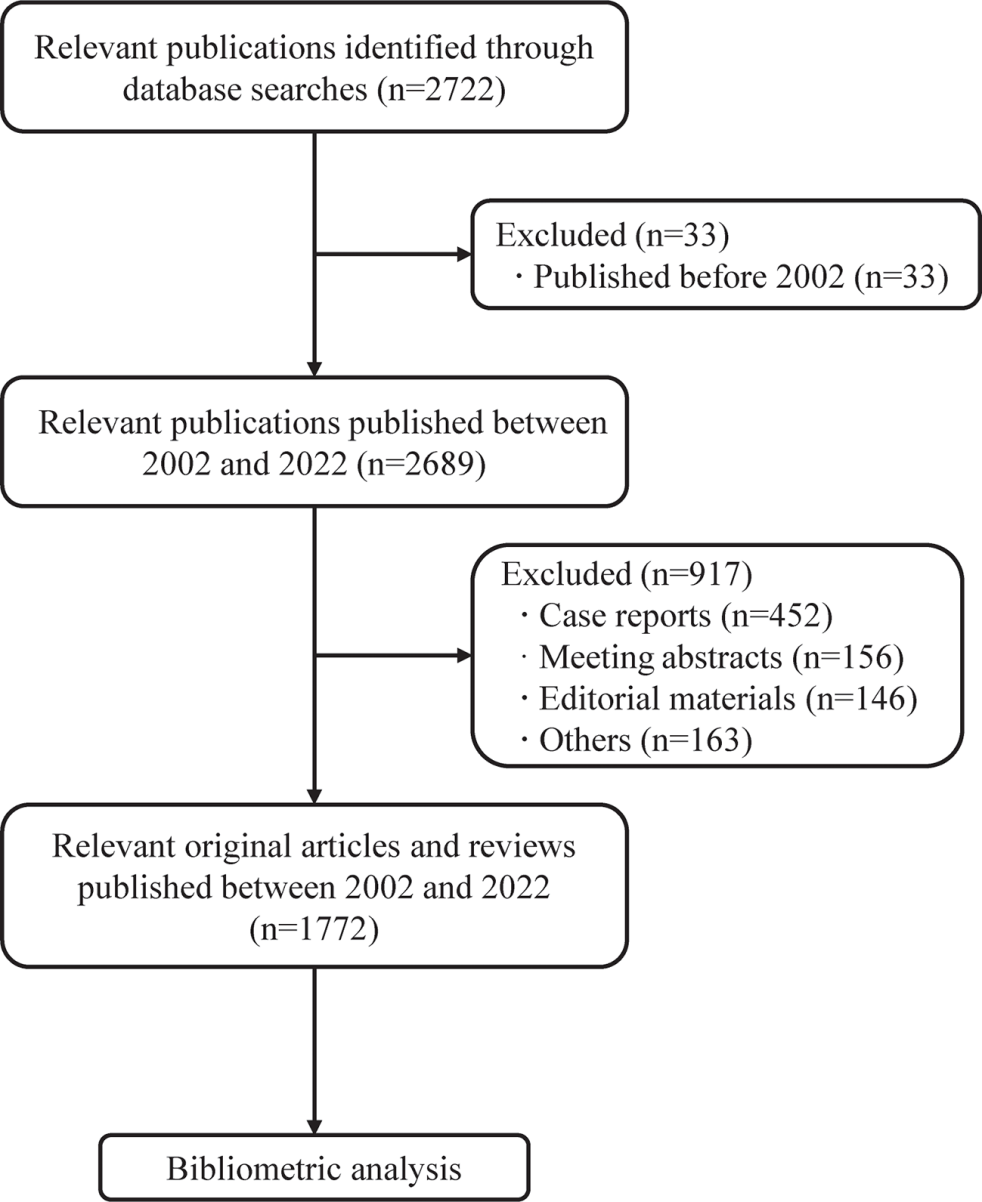
The inclusion and exclusion of publications on AIP.

**Figure 2:**
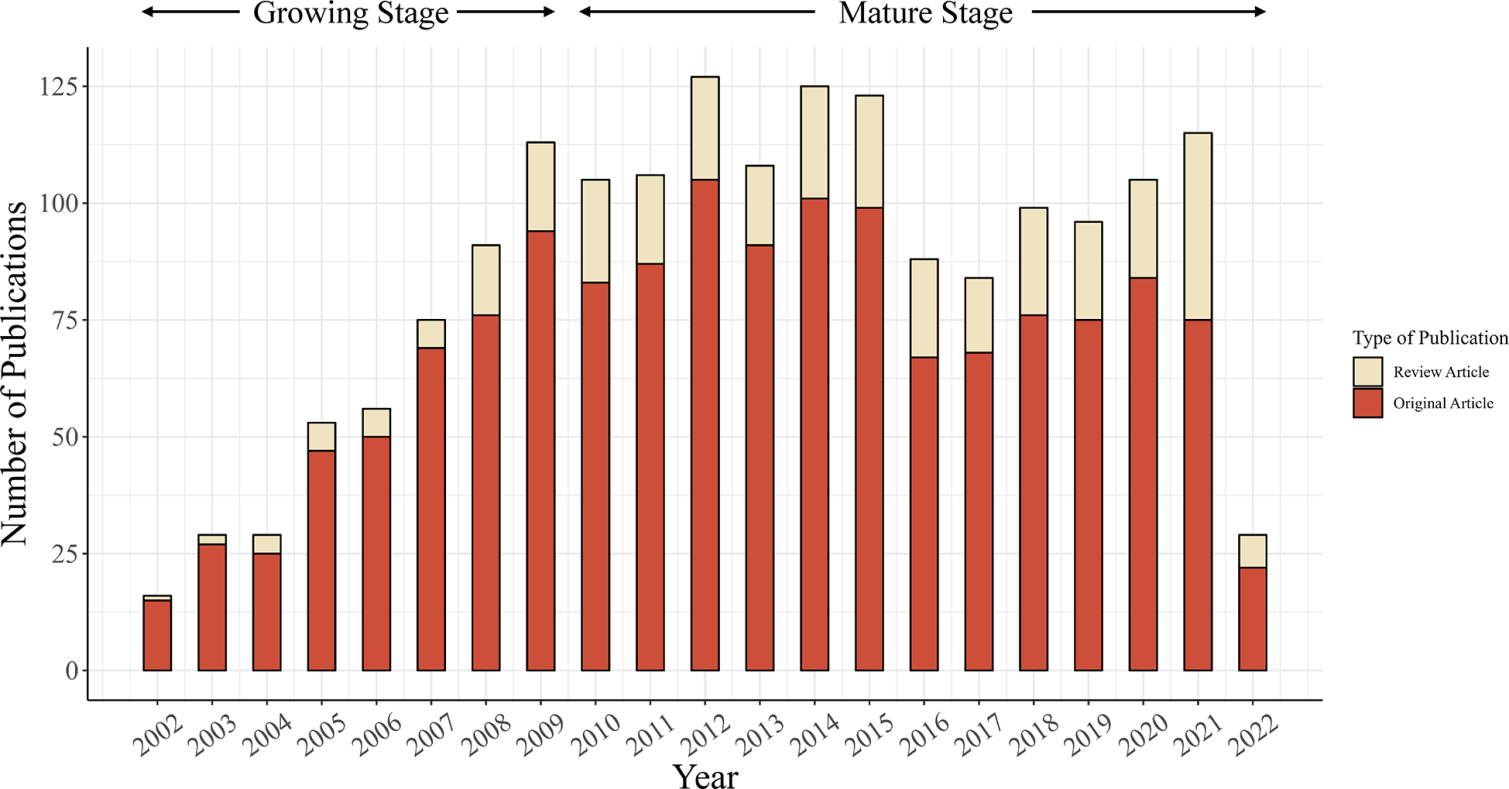
Annual number of publications on AIP.

Overall, 1,772 publications related to AIP published between 2002 and 2022 were found in the WoSCC, including 1,436 original articles and 336 review articles. **Figure 1** shows the inclusion and exclusion of publications. Most identified literatures were published in English (n=1,689, 95.3%), followed by German (n=36, 2.0%), Spanish (n=18, 1.0%), French (n=12, 0.7%), Russian (n=4, 0.2%), and other 7 languages. **Figure 2** presents the annual number of publications on AIP. According to the annual output, we artificially divided this period into two stages: the growing stage (2002–2009) and the mature stage (2010–2022). The yearly number of publications increased from 16 in 2002 to 113 in 2009, with an average increase of 13.9 publications per year. During the mature stage, the annual output stayed at >80 per year, and the highest output was 127 in 2012. The ratios of original and review articles for each year are displayed in **Supplementary Table S1**. Notably, the ratio of review articles increased from 6.3% in 2002 to 34.8% in 2021. So far, this collection of articles has been cited 55,504 times, with an average of 27.29 citations per article.

### 3.2 Leading countries/regions

**Figure 3:**
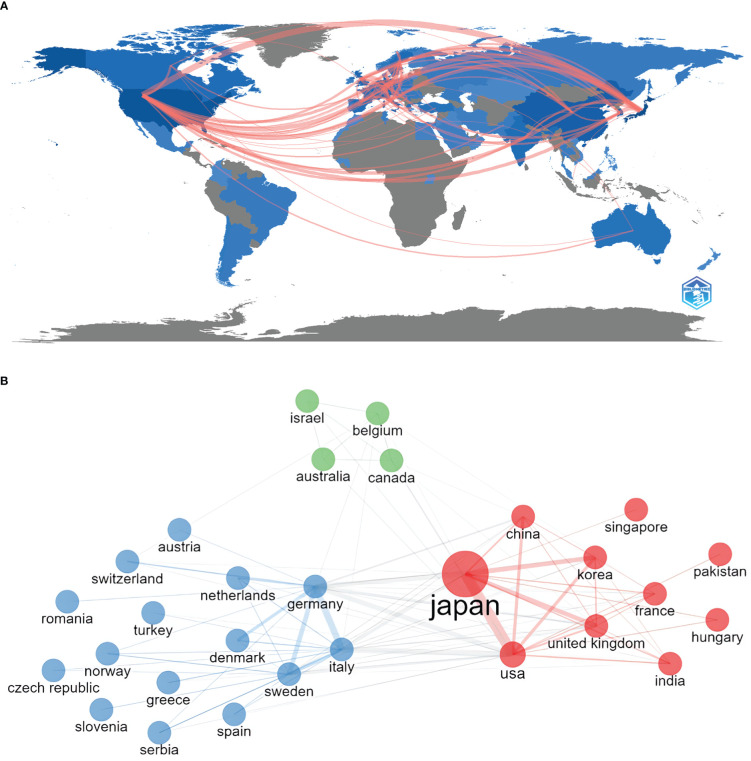
Leading Countries/Regions. **(A)** Distribution of publications and collaborations between countries/regions and **(B)** clustering of collaboration among countries/regions.

Between 2002 and 2022, 63 countries/regions over 6 continents published articles on AIP, with close collaboration among East Asia, North America, and Western Europe (**Figure 3A**).

The top 10 most productive countries are listed in **Table 1**. Japan was the most productive country with respect to AIP research (n=728, 41.1%), followed by the United States (n=336, 19%), Germany (n=147, 8.3%), China (n=127, 7%), and Italy (n=107, 6%). While articles from Japan received the most total citations (29,705 times), those from the United States showed the highest number of average citations (48.47 times per article). Three clusters of collaboration were identified (**Figure 3B**). Active collaborations were noted among Japan, the United States, China, and South Korea, whereas Germany had close collaboration with Italy. Collaborative centrality measures the position of a country/institution/author in the network of research collaboration, and a higher level of collaborative centrality reflects a greater number of research connections with partners. The United States showed the highest level of collaborative centrality, followed by Japan and Germany in this study.

**Table 1:** The top 10 most productive countries in terms of AIP research.

Rank	Country	Publications n (%)	Total citations	Average citations	Collaborative centrality
1	Japan	728 (41.1%)	29,705	40.80	0.12
2	United States	336 (19%)	16,285	48.47	0.3
3	Germany	147 (8.3%)	4,649	31.63	0.09
4	China	124 (7.0%)	1,996	16.10	0.05
5	Italy	107 (6.0%)	4,736	44.26	0.08
6	South Korea	89 (5.0%)	3,755	42.19	0.01
7	United Kingdom	71 (4.0%)	2,803	39.48	0.06
8	France	39 (2.2%)	1,024	26.26	0.03
9	Sweden	39 (2.2%)	2,007	51.46	0.08
10	Netherlands	34 (1.9%)	1,570	46.18	0.06

### 3.3 Active institutions and authors

**Figure 4:**
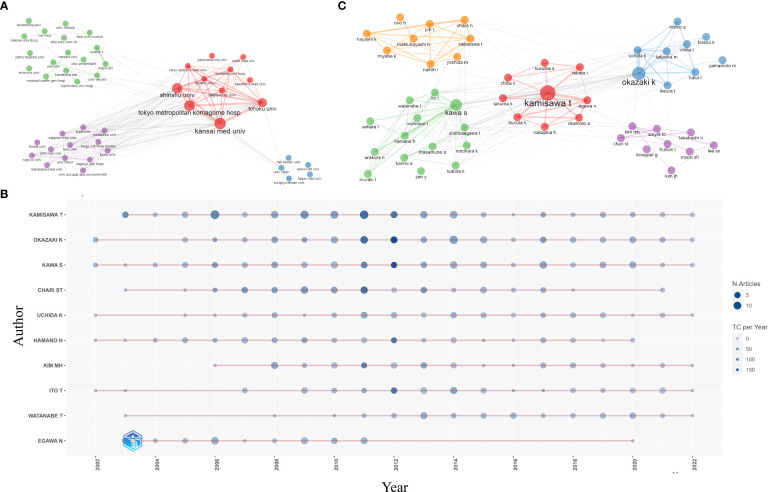
Active institutions and authors. **(A)** Clustering of collaboration among institutions **(B)** annual output of the top 10 most productive authors and **(C)** clustering of collaboration among authors.

A total of 6,767 authors from 1,617 institutions published articles on AIP. The top 10 most productive institutions are listed in **Table 2**. Tokyo Metropolitan Komagome Hospital (n=112, 6.3%) was the leading institution, followed by Kansai Medical University (n=100, 5.6%), Mayo Clinic (n=95, 5.4%), Shinshu University (n=95, 5.4%), and the University of Ulsan (n=57, 3.2%). Seven out of the ten most productive institutions were located in Japan. Four clusters of collaboration among institutions were identified (**Figure 4A**). The cluster led by Tokyo Metropolitan Komagome Hospital, Kansai Medical University, Shinshu University, Tohoku University, and Nagoya City University showed the closest cooperation. Mayo Clinic displayed the highest level of collaborative centrality, followed by Tokyo Metropolitan Komagome Hospital and the University of Verona.

**Table 2:** The top 10 most productive institutions in terms of AIP research.

Rank	Institution	Country	Publications n (%)	Total citations	Average citations	Collaborative centrality
1	Tokyo Metropolitan Komagome Hospital	Japan	112 (6.3%)	9,599	85.71	0.08
2	Kansai Medical University	Japan	100 (5.6%)	7,858	78.58	0.04
3	Mayo Clinic	United States	95 (5.4%)	7,923	83.4	0.09
4	Shinshu University	Japan	95 (5.4%)	8,241	86.75	0.05
5	University of Ulsan	South Korea	57 (3.2%)	3,163	55.49	0.04
6	Tohoku University	Japan	55 (3.1%)	5,588	101.6	0.03
7	Nagoya City University	Japan	53 (3.0%)	3,610	68.11	0.03
8	University of Verona	Italy	52 (2.9%)	3,561	68.48	0.08
9	Kanazawa University	Japan	50 (2.8%)	4,822	96.44	0.06
10	Kyoto University	Japan	41 (2.3%)	3,437	83.83	0.02

The top three most prolific authors were Terumi Kamisawa from Tokyo Metropolitan Komagome Hospital (n=117, 6.6%), Kazuichi Okazaki from Kansai Medical University (n=103, 5.8%), and Shigeyuki Kawa from Matsumoto Dental University (n=94, 5.3%) (**Table 3**). Eight out of the ten most productive authors came from Japan, one was from Korea, and another was from the United States. Suresh T. Chari of the University of Texas MD Anderson Cancer Center showed the highest H-index of 87. **Figure 4B** displays the annual output of the top 10 most productive authors. Cooperation among authors was relatively close, with five clusters present (**Figure 4C**). Terumi Kamisawa showed the highest level of collaborative centrality, followed by Suresh T Chari and Myung-Hwan Kim.

**Table 3:** The top 10 most productive authors in terms of AIP research.

Rank	Author	Institution	Country	Publications n (%)	Total citations	Average citations	H-index	Collaborative centrality
1	Terumi Kamisawa	Tokyo Metropolitan Komagome Hospital	Japan	117 (6.6%)	9,720	83.08	60	0.18
2	Kazuichi Okazaki	Kansai Medical University	Japan	103 (5.8%)	8,296	80.54	60	0.04
3	Shigeyuki Kawa	Matsumoto Dental University	Japan	94 (5.3%)	7,773	82.69	57	0.02
4	Suresh T Chari	University of Texas MD Anderson Cancer Center	United States	62 (3.5%)	6,989	112.73	87	0.09
5	Kazushige Uchida	Kansai Medical University	Japan	61 (3.4%)	2,115	34.67	40	0.03
6	Hideaki Hamano	Shinshu University	Japan	59 (3.3%)	4,537	76.90	40	0.01
7	Myung-Hwan Kim	University of Ulsan	Korea	51 (2.9%)	3,093	60.65	66	0.09
8	Tetsuhide Ito	Fukuoka Sanno Hospital	Japan	51 (2.9%)	3,970	77.84	41	0.01
9	Tomohiro Watanabe	Kindai University	Japan	45 (2.5%)	1,181	26.24	37	0.03
10	Naoto Egawa	Tokyo Metropolitan Komagome Hospital	Japan	43 (2.4%)	3,467	80.62	37	0.03

### 3.4 Core journals and references

**Figure 5:**
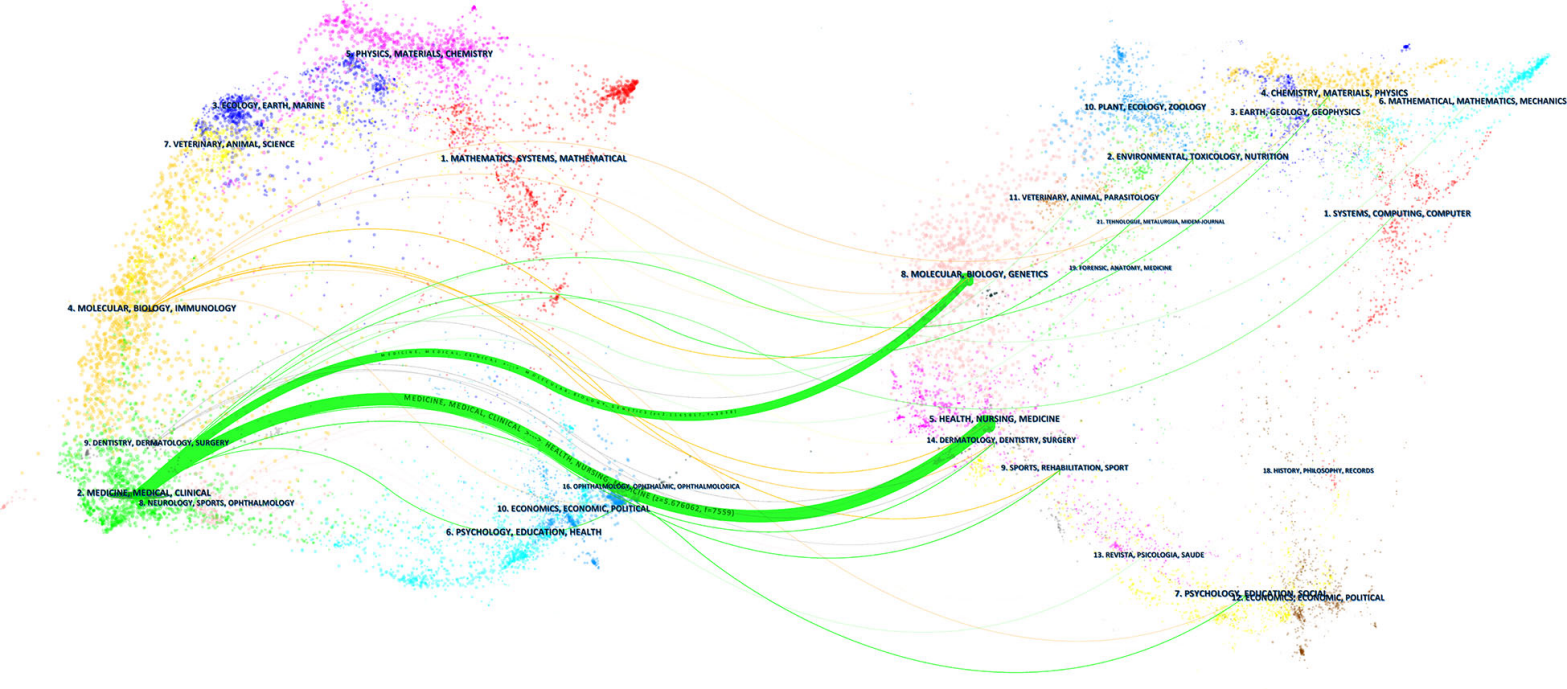
The dual-map overlay of journals publishing studies on AIP. Citing journals are on the left, cited journals are on the right, and lines represent the citation relationship.

Overall, 501 journals published studies on AIP. The top 10 most productive journals with respect to AIP research are summarized in **Table 4**. *Pancreas* was the most productive journal (n=95, 5.4%), followed by the *Journal of Gastroenterology* (n=67, 3.8%), *Internal Medicine* (n=66, 3.7%), *Pancreatology* (n=63, 3.6%), and *World Journal of Gastroenterolog*y (n=62, 3.5%). Publications from the *American Journal of Gastroenterology* had the highest number of average citations (104 times per article). The dual-map overlay revealed multiple inter-domain connections between journals (**Figure 5**). In **Figure 5**, the journals on the left are the citing journals, whereas the journals on the right are the cited journals; the lines denote the citation relationship between them (4). Two main citation paths were identified. Publications in the journals of Health/Nursing/Medicine and Molecular/Biology/Genetics were mostly cited by publications in the journals of Medicine/Medical/Clinical.

**Table 4:** The top 10 core journals in terms of AIP research.

Rank	Journal	Publications n (%)	Total citations	Average citations	2021 JCR category quartile	2021 IF
1	*Pancreas*	96 (5.4%)	4,601	47.93	Q3	3.243
2	*Journal of Gastroenterology*	67 (3.8%)	5,416	80.84	Q2	6.772
3	*Internal Medicine*	66 (3.7%)	1,384	20.97	Q4	1.282
4	*Pancreatology*	63 (3.6%)	1,456	23.11	Q3	3.977
5	*World Journal of Gastroenterology*	62 (3.5%)	1,126	18.16	Q2	5.374
6	*Journal of the Pancreas*	41 (2.3%)	438	10.68	/	/
7	*Clinical Journal of Gastroenterology*	29 (1.6%)	100	3.45	/	/
8	*Gastrointestinal Endoscopy*	26 (1.5%)	1,934	74.38	Q1	10.396
9	*American Journal of Gastroenterology*	25 (1.4%)	2,610	104.40	Q1	12.045
10	*American Journal of Surgical Pathology*	25 (1.4%)	2,495	99.80	Q1	6.298

JCR, Journal Citation Report; and IF, impactor factor.

The symbol "/" means that “Journal of the Pancreas” and “Clinical Journal of Gastroenterology” do not have “2021 JCR category quartile” and “2021 IF”.

The top 10 references with the highest number of citations are presented in **Table 5**. In 2003, Kamisawa et al. suggested that AIP could be a pancreatic manifestation of a chronic fibroinflammatory condition currently known as IgG4-RD. In 2009, Kamisawa et al. proposed the standard steroid regimen for AIP. In 2011, Shimosegawa et al. developed the International Consensus Diagnostic Criteria (ICDC) for AIP, which is the diagnostic standard most frequently used in clinical practice and categorizes AIP into types 1 and 2. In summary, the top 10 core references mainly focused on the diagnosis and treatment of AIP.

**Table 5:** The top 10 references on AIP with the highest number of citations.

Rank	First author	Title	Journal	Year of publication	Total citations
1	Hisanori Umehara	Comprehensive diagnostic criteria for IgG4-related disease (IgG4-RD), 2011	*Modern Rheumatology*	2012	1,181
2	Terumi Kamisawa	A new clinicopathological entity of IgG4-related autoimmune disease	*Journal of Gastroenterology*	2003	902
3	Tooru Shimosegawa	International Consensus Diagnostic Criteria for Autoimmune Pancreatitis Guidelines of the International Association of Pancreatology	*Pancreas*	2011	850
4	Suresh T Chari	Diagnosis of autoimmune pancreatitis: The Mayo Clinic experience	*Clinical Gastroenterology and Hepatology*	2006	678
5	Hisanori Umehara	A novel clinical entity, IgG4-related disease (IgG4RD): general concept and details	*Modern Rheumatology*	2012	566
6	Amaar Ghazale	Immunoglobulin G4-associated cholangitis: Clinical profile and response to therapy	*Gastroenterology*	2008	557
7	Rob C Aalberse	Immunoglobulin G4: an odd antibody	*Clinical & Experimental Allergy*	2009	529
8	Kenji Notohara	Idiopathic chronic pancreatitis with periductal lymphoplasmacytic infiltration: clinicopathologic features of 35 cases	*The American Journal of Surgical Pathology*	2003	510
9	Terumi Kamisawa	Standard steroid treatment for autoimmune pancreatitis	*Gut*	2009	442
10	Giuseppe Zamboni	Histopathological features of diagnostic and clinical relevance in autoimmune pancreatitis: a study on 53 resection specimens and 9 biopsy specimens	*Virchows Archive*	2004	429

### 3.5 Analysis of keywords

**Figure 6:**
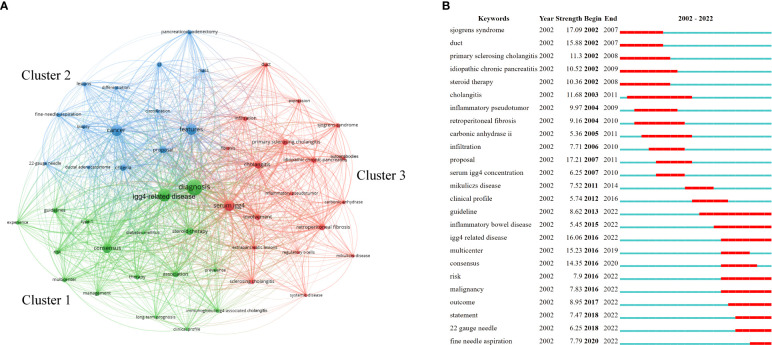
Analysis of keywords related to AIP. **(A)** Clustering of the top 50 keywords with the highest number of occurrences and **(B)** keyword burst detection of the top 25 keywords with the strongest emergent strength.

The top 10 keywords with the highest number of occurrences are listed in **Table 6**. “Diagnosis” was the most mentioned keyword. Among the top 50 keywords, three clusters were identified according to keyword co-occurrence (i.e., how frequently two keywords appear in the same literature), as shown in **Figure 6A**. The cluster led by “diagnosis” and “IgG4-related disease” displayed the highest number of occurrences, followed by the cluster led by “cancer” and “features” and then the cluster led by “serum IgG4” and “cholangitis.”

**Table 6:** The top 10 most common keywords.

Rank	Keyword	Cluster	Occurrence
1	diagnosis	1	513
2	IgG4-related disease	1	358
3	cancer	2	286
4	features	2	277
5	serum IgG4	3	265
6	consensus	1	214
7	cholangitis	3	150
8	proposal	2	134
9	steroid-therapy	1	129
10	retroperitoneal fibrosis	3	109

Keyword burst detection is regarded as an indicator of research frontiers or emerging topics in a specific field over time (9, 10). The top 25 keyword terms with the strongest emergent strength are illustrated in **Figure 6B**. In **Figure 6B**, “Year” indicates the year in which the keyword first appeared; “Begin” and “End” indicate the starting and ending years of the keyword as a frontier, respectively; and “Strength” indicates the emergent strength. **Figure 6B** reflects the research frontiers during different time periods. “Proposal” was the focus of research in 2007–2011, with 17.21 being the strongest emergent strength. “Risk,” “malignancy,” “outcome,” “22-gauge needle,” and “fine-needle aspiration” have been the research frontiers in recent years.

## 4 Discussion

**Figure 7:**
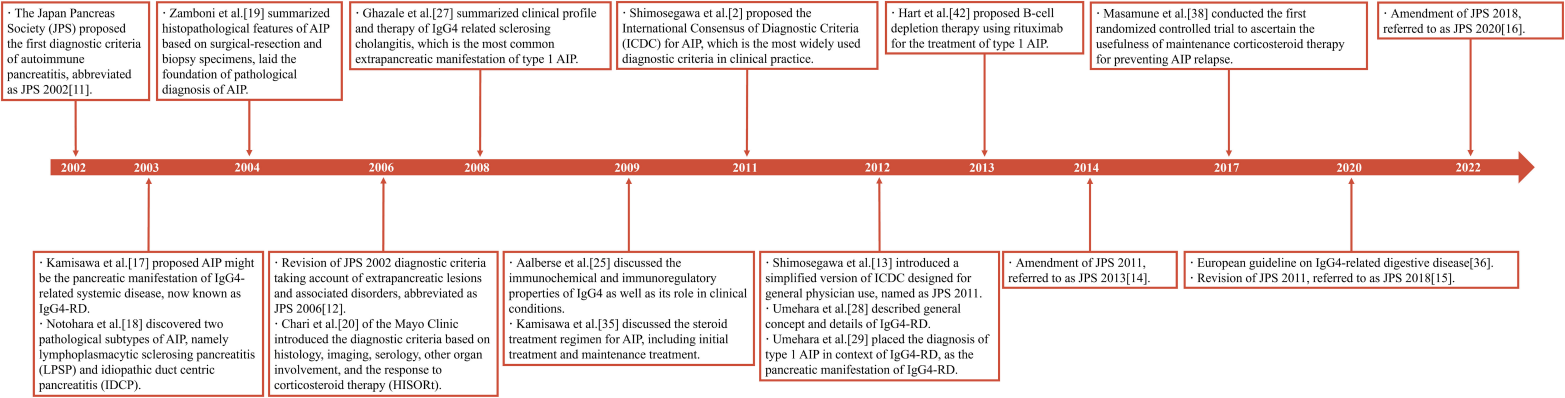
Timeline of some landmark publications of AIP.

Herein, we conducted a bibliometric analysis of AIP-related publications over the last 20 years. The annual number of publications showed an upward trend from 2002 to 2009 and has remained relatively stable since 2010. Leading countries/regions, active institutions and authors, core journals and references, and keywords were evaluated. Some landmark articles were identified (**Figure 7**). To our knowledge, this is the first bibliometric analysis of AIP reported.

Japan was the leading country with respect to AIP research, contributing over 40% of studies on AIP. Seven out of the ten most productive institutions and eight out of the ten most productive authors were from Japan. Furthermore, six out of the top 10 most cited articles were first authored by Japanese researchers. Collaboration among Tokyo Metropolitan Komagome Hospital, Kansai Medical University, Shinshu University, Tohoku University, and Nagoya City University was relatively close. The Japan Pancreas Society had provided timely updates on the diagnostic criteria for AIP (11–16).

Diagnosis of AIP is currently the research focus. The Japan Pancreas Society proposed the first diagnostic criteria in 2002 (11). Since then, much progress has been made. Kamisawa et al. (17) proposed that AIP might be a pancreatic manifestation of a chronic fibroinflammatory condition currently known as IgG4-RD. Notohara et al. (18) and Zamboni et al. (19) summarized the histopathological findings of AIP and reported two subtypes—namely, lymphoplasmacytic sclerosing pancreatitis and idiopathic duct-centric pancreatitis. In 2006, Chari et al. (20) introduced the HISORt diagnostic criteria, which are based on histology, imaging of the pancreas using computed tomography or magnetic resonance imaging, serum IgG4 levels, other organ involvement, and response to steroid therapy. The integration of histology, radiology, serology, and follow-up formed a pathway for the diagnosis of AIP, which remained in further studies. In 2011, Shimosegawa et al. (2) proposed the ICDC, which are the most widely used diagnostic criteria in clinical practice. According to the ICDC, AIP can be subclassified into types 1 and 2 (2). AIP types 1 and 2 share similar radiological findings such as sausage-like pancreatic enlargement, rim-like enhancement around the lesion, delayed homogenous enhancement in the pancreatic parenchyma, and long or multiple strictures without marked upstream dilatation in the main pancreatic duct (21–23). However, AIP types 1 and 2 differ in terms of serology and histopathology. As the pancreatic manifestation of IgG4-RD, type 1 AIP exhibits elevated serum IgG4 levels (3, 24, 25). Histopathologically, type 1 AIP corresponds to lymphoplasmacytic sclerosing pancreatitis, with abundant IgG4-positive plasma cell infiltration (2). Other organ involvement, including sclerosing cholangitis, retroperitoneal fibrosis, and sclerosing sialadenitis, is common in type 1 AIP (26–29), with IgG4-related sclerosing cholangitis being the most common organ involvement, occurring in up to 80% of type 1 AIP cases (30, 31). In contrast, other organ involvement is less common in type 2 AIP (26). Type 2 AIP accounts for <5% of AIP cases in Eastern countries and is more common in Western countries, accounting for up to 10%–20% of AIP cases in Western countries (32, 33). Approximately 30% of type 2 AIP cases are estimated to be associated with inflammatory bowel disease, particularly ulcerative colitis (2). Type 2 AIP has an earlier onset (in individuals in their 30s) and a lower incidence of disease relapse after initial induction of remission than type 1 AIP (32). Histopathologically, type 2 AIP corresponds to idiopathic duct-centric pancreatitis and usually exhibits infiltration of no or very few IgG4-positive plasma cells (2). Serum IgG4 levels are often within the normal range (3). Owing to the paucity of reliable serum biomarkers, the diagnosis of type 2 AIP heavily relies on pancreatic histopathology (26). Moreover, both types of AIP share a dramatic response to glucocorticoid therapy (32).

Much progress has been made in the treatment of AIP. Glucocorticoids are the first-line therapy. Indications for glucocorticoid therapy are symptoms such as obstructive jaundice, abdominal pain, and other organ involvement (34). As for the induction of remission, Kamisawa et al. (35) recommended an initial dose of 0.6 mg/kg/day of oral prednisolone for 2–4 weeks. Symptoms are anticipated to be relieved within days after commencing the treatment (36). Assessment of the response to initial treatment with biochemical, serological, and radiological work-up at weeks 2–4 is recommended (36). Subsequently, glucocorticoids should be gradually tapered off, usually 5 mg every 1–2 weeks. When glucocorticoid therapy fails to relieve AIP-related symptoms, a reevaluation of diagnosis should be in order (15, 16). Clinicians should be particularly cautious about pancreatic cancer misdiagnoses. There are disputes over glucocorticoid maintenance therapy. In Western countries, glucocorticoid therapy is generally limited to the induction of remission without maintenance (37) because prolonged administration may increase the risk of infections, diabetes, osteoporosis, and cataracts (34). However, Masamune et al. (38) conducted the first AIP-related randomized controlled trial, the results of which favored maintenance therapy. Maintenance therapy with prednisolone at a dose of 5–7.5 mg/day was continued for 3 years. Compared with the cessation group, which had withdrawn at 26 weeks since the initial glucocorticoid therapy, the maintenance group achieved better 3-year relapse-free survival (42.1% vs. 76.7%, p=0.007) (38). Moreover, no major glucocorticoid-related complications requiring treatment cessation were found in the maintenance group (38). Thus, the Japanese consensus guidelines advocate a 3-year maintenance therapy to prevent disease relapse (16). Immunosuppressants such as azathioprine, methotrexate, and mycophenolate mofetil may be beneficial for patients with AIP. A recent meta-analysis had suggested that azathioprine was effective in preventing AIP relapse (39). B-cell depletion therapy has been proposed as a treatment for recurrent type 1 AIP. CD20 is a B-cell surface marker involved in calcium channel activation, cell proliferation, and B-cell differentiation (40). Rituximab is a monoclonal antibody targeting human CD20. Rituximab can induce complement activation and cell-mediated cytotoxicity, leading to B-cell depletion (41). Hart et al. (42) reported rituximab as a treatment for recurrent AIP. A decrease in serum IgG4 concentration and the extinction of pancreatic hypermetabolic signal on positron emission tomography were achieved in type 1 AIP after rituximab treatment (43). Other therapies for type 1 AIP, including rilzabrutinib (Bruton tyrosine kinase inhibitor), belimumab (B-cell activating factor inhibitor), and inebilizumab (anti-CD19 monoclonal antibody), are under investigation (44).

Keyword burst detection is capable of tracing research frontiers (9, 10). Some emerging topics had been recognized, including “risk,” “malignancy,” and “outcome.” Patients with AIP are at a high risk for malignancy (45). A recent meta-analysis of 17 studies involving 2,746 patients revealed that the overall prevalence of malignancy in patients with AIP was 9.6% (46). The top 5 most prevalent malignancies in patients with AIP were gastric, colorectal, bladder, prostate, and pancreatic cancers (46). The majority of pancreatic cancer cases in patients with AIP occurred at no less than 2 years after an AIP diagnosis (47). Other than malignancy, the long-term outcomes of AIP include diabetes mellitus (DM) and pancreatic exocrine insufficiency (PEI) (48, 49). Chronic inflammation in patients with AIP may cause damage to the pancreatic β-cells and acinar cells, leading to DM and PEI (50). By alleviating the inflammation and swelling of pancreatic tissues with glucocorticoid therapy, both endocrine and exocrine functions are supposed to be restored (50, 51). However, DM and PEI are often described at the time of diagnosis and during follow-up. The pooled prevalence of DM and PEI in patients with AIP at the time of diagnosis is 36.5% and 45.2%, respectively (52). Moreover, the pooled prevalence of DM during follow-up is 40.9% (52). The prevalence of PEI during follow-up ranged from 23.8% to 72.7% (49, 53, 54). Studies concerning malignancy, DM, and PEI in patients with AIP are mostly retrospective. More high-quality prospective cohort studies are required to better understand the long-term outcomes of patients with AIP.

In addition to clinical outcomes, pancreatic tissue acquisition is now gaining attention. A “22-gauge needle” and “fine-needle aspiration” have been recognized as research hotspots since the late 2010s by keyword burst analysis. Endoscopic ultrasound-guided fine-needle aspiration (EUS-FNA) and endoscopic ultrasound-guided fine-needle biopsy (EUS-FNB) have been utilized for pancreatic tissue acquisition in patients with AIP. When pathology specimen collection is necessary for diagnosis or when malignancy is suspected, EUS-FNA or EUS-FNB should be taken into consideration (16). EUS-FNA is designed for the aspiration of cells from the target lesion using a conventional straight needle. Tissue core samples acquired by EUS-FNA are usually limited in size, making the histopathological diagnosis of AIP less than satisfactory. With the help of recently developed core needles, EUS-FNB is capable of obtaining a large amount of tissue core samples with preserved tissue architecture (16, 55). According to the ICDC, the histological findings of AIP can be categorized into levels 1 and 2 (2). A recent meta-analysis demonstrated that EUS-FNB had better diagnostic yield than EUS-FNA (56). The pooled diagnostic yield for level 1 and 2 histological findings was 55.8% for EUS-FNA and 87.2% for EUS-FNB (p=0.03) (56). As for the needle size, a 19-gauge needle exhibited a better pooled diagnostic yield for level 1 and 2 histological findings than a 22-gauge needle (88.9% *vs*. 60.6%, p=0.023) (56). However, studies investigating the diagnostic yield of EUS-FNA or EUS-FNB had mainly focused on type 1 AIP, rather than type 2 AIP, possibly because these studies were largely conducted in Eastern countries where type 2 AIP is quite rare (<5% of total AIP cases) (32, 33). Moreover, histological diagnosis is more essential for type 2 AIP than for type 1 AIP, as there are no reliable serological markers. Further studies should recruit more patients with type 2 AIP by inviting more European and American centers.

Research cooperation among countries/regions, institutions and authors has been identified by the cooperation network in our study. Active collaborations were noted among Japan, the United States, China, and South Korea. Due to the limited cases of type 2 AIP reported, its genetic predisposition, relationship with inflammatory bowel disease, and long-term outcome have not been characterized in detail (57). This might be because the current knowledge base of AIP is mainly generated from Eastern-population-driven information, especially in Japan, where type 2 AIP is less common. Therefore, further collaborative global research is essential to understand AIP comprehensively.

This study has some limitations. First, the data were retrieved exclusively from the WoSCC, rather than other databases such as Embase and MEDLINE. The WoSCC is the most commonly applied database in bibliometric analysis because it provides timely and comprehensive updates on citation network. Moreover, software that were applied in our bibliometric analysis had difficulties in integrating data from different resources. Second, the increase in the number of publications on AIP from 2002 to 2009 might have resulted from the overall increase in scientific outputs in the medical field over the past two decades. Finally, some new emerging topics related to AIP might not have been identified because of the sensitivity of algorithms applied in the analysis. Although multiple software/packages have been used in our study, the information provided in our study is still constricted by algorithms applied in bibliometric analysis. Further development in the methodology of bibliometric analysis might be helpful in resolving these limitations.

## 5 Conclusion

Over the past two decades, Japan was the leading country in AIP research, with more than half of the top 10 most productive institutions and top 10 most productive authors being from Japan. Research papers were mainly published in specialized journals. Diagnosis of AIP was the research focus. Long-term outcomes and pancreatic tissue acquisition are recognized as research frontiers for AIP.

## Data availability statement

The raw data supporting the conclusions of this article will be made available by the authors, without undue reservation.

## Author contributions

X-DZ: conceptualization, methodology, software, formal analysis, resources, data curation, visualization, and writing – original draft. YZ: methodology, software, validation, formal analysis, resources, data curation, visualization, and writing – original draft. Y-ZZ: methodology, software, validation, formal analysis, resources, data curation, visualization, and writing – original draft. C-HZ: conceptualization, supervision, funding acquisition, and writing – review and editing. D-WZ: conceptualization, supervision, project administration, and writing – review and editing. All authors contributed to the article and approved the submitted version.
