# Supplementary material for: Autoimmune pancreatitis: A bibliometric analysis from 2002 to 2022
Source: Front Immunol. 2023 Feb 22;14:1135096. doi: 10.3389/fimmu.2023.1135096 (PMC9992966; doi:10.3389/fimmu.2023.1135096)
Supplement: Supplementary file 1 [file Table_1.docx]

Supplementary Material

**Table S1.** The ratios of original and review articles for each year.

| **Year** | **Original article**  **n (%)** | **Review article**  **n (%)** | **Total** |
| --- | --- | --- | --- |
| 2002 | 15 (93.8) | 1 (6.3) | 16 |
| 2003 | 27 (93.1) | 2 (6.9) | 29 |
| 2004 | 25 (86.2) | 4 (13.8) | 29 |
| 2005 | 47 (88.7) | 6 (11.3) | 53 |
| 2006 | 50 (89.3) | 6 (10.7) | 56 |
| 2007 | 69 (92.0) | 6 (8.0) | 75 |
| 2008 | 76 (83.5) | 15 (16.5) | 91 |
| 2009 | 94 (83.2) | 19 (16.8) | 113 |
| 2010 | 83 (79.0) | 22 (21.0) | 105 |
| 2011 | 87 (82.1) | 19 (17.9) | 106 |
| 2012 | 105 (82.7) | 22 (17.3) | 127 |
| 2013 | 91 (84.3) | 17 (15.7) | 108 |
| 2014 | 101 (80.8) | 24 (19.2) | 125 |
| 2015 | 99 (80.5) | 24 (19.5) | 123 |
| 2016 | 67 (76.1) | 21 (23.9) | 88 |
| 2017 | 68 (81.0) | 16 (19.0) | 84 |
| 2018 | 76 (76.8) | 23 (23.2) | 99 |
| 2019 | 75 (78.1) | 21 (21.9) | 96 |
| 2020 | 84 (80.0) | 21 (20.0) | 105 |
| 2021 | 75 (65.2) | 40 (34.8) | 115 |
| 2022 | 22 (75.9) | 7 (24.1) | 29 |
